# Global transcriptome profiles of *Camellia sinensis* during cold acclimation

**DOI:** 10.1186/1471-2164-14-415

**Published:** 2013-06-22

**Authors:** Xin-Chao Wang, Qiong-Yi Zhao, Chun-Lei Ma, Zong-Hong Zhang, Hong-Li Cao, Yi-Meng Kong, Chuan Yue, Xin-Yuan Hao, Liang Chen, Jian-Qiang Ma, Ji-Qiang Jin, Xuan Li, Ya-Jun Yang

**Affiliations:** 1 Tea Research Institute, Chinese Academy of Agricultural Sciences; National Center for Tea Improvement, Key Laboratory of Tea Plant Biology and Resources Utilization, Ministry of Agriculture, Hangzhou 310008, China; 2 Key Laboratory of Synthetic Biology, Institute of Plant Physiology and Ecology, Shanghai Institutes for Biological Sciences Chinese Academy of Sciences, Shanghai 200032, China; 3 The University of Queensland, Queensland Brain Institute, Qld 4072, Australia

**Keywords:** *Camellia Sinensis*, Cold Acclimation, RNA-Seq, DGE, Genome-wide Expression Profiles, Tea Plants

## Abstract

**Background:**

Tea is the most popular non-alcoholic health beverage in the world. The tea plant (*Camellia sinensis* (L.) O. Kuntze) needs to undergo a cold acclimation process to enhance its freezing tolerance in winter. Changes that occur at the molecular level in response to low temperatures are poorly understood in tea plants. To elucidate the molecular mechanisms of cold acclimation, we employed RNA-Seq and digital gene expression (DGE) technologies to the study of genome-wide expression profiles during cold acclimation in tea plants.

**Results:**

Using the Illumina sequencing platform, we obtained approximately 57.35 million RNA-Seq reads. These reads were assembled into 216,831 transcripts, with an average length of 356 bp and an N50 of 529 bp. In total, 1,770 differentially expressed transcripts were identified, of which 1,168 were up-regulated and 602 down-regulated. These include a group of cold sensor or signal transduction genes, cold-responsive transcription factor genes, plasma membrane stabilization related genes, osmosensing-responsive genes, and detoxification enzyme genes. DGE and quantitative RT-PCR analysis further confirmed the results from RNA-Seq analysis. Pathway analysis indicated that the “carbohydrate metabolism pathway” and the “calcium signaling pathway” might play a vital role in tea plants’ responses to cold stress.

**Conclusions:**

Our study presents a global survey of transcriptome profiles of tea plants in response to low, non-freezing temperatures and yields insights into the molecular mechanisms of tea plants during the cold acclimation process. It could also serve as a valuable resource for relevant research on cold-tolerance and help to explore the cold-related genes in improving the understanding of low-temperature tolerance and plant-environment interactions.

## Background

Low temperatures are one of the most important environmental factors that temperate plants have to cope with during their life cycles. Some plants can enhance their freezing tolerance after exposure to low but non-freezing temperatures for a period of time, a process known as cold acclimation (CA) [1]. CA is a complex process that involves cellular, physiological, metabolic and molecular modifications. When plants sense the cold temperature, a series of protective mechanisms are triggered [2-4]. These include resetting the cellular framework; alternating the composition, structure and function of the plasma membrane; synthesizing cryoprotectant molecules such as soluble sugars, sugar alcohols and low-molecular-weight nitrogenous compounds; decreasing the ratio of free water content; improving the scavenging activity of reactive oxygen species (ROS); and introducing antifreeze proteins. These alterations help plants maintain a metabolic balance of substance and energy in cold environments. A group of cold-related genes has been reported to regulate these aforementioned changes [2-7]. Moreover, changes in gene expression have been demonstrated to occur during CA in a wide range of plant species, and hundreds of cold inducible genes have been identified [8].

Tea is the most popular non-alcoholic health beverage in the world, and the tea plant (*Camellia sinensis* (L.) O. Kuntze) is one of the most important economic crops in China, India, Sri Lanka, Kenya, among others [9]. As an evergreen woody plant, the tea plant can be grown in tropical to subtropical climates. Due to the local climate changes, tea plants have to cope with low temperatures during the wintertime. Low temperatures are one of the most critical environmental factors that limit its growth, survival and geographical distribution [10]. Thus, finding ways to improve tea plants’ resistance to low temperatures is of great importance. Like other perennial evergreen woody crops, during the CA process, the cold tolerance of tea plants enhances with the decrease in temperature and reduces with the increase in temperature. A previous study showed that when the average air temperature decreases to around 7°C, tea plants undergo the CA process, and after the average air temperature increases to over 9°C, tea plants start the de-acclimation process [11]. There are few studies that have focused on the cellular, physiological and metabolic changes during CA in tea plants. When tea plants undergo the CA process, the thickness of palisade tissue is increased and the stability of plasma membrane is enhanced. In addition, the concentration of the cytochylema and ratio of bound water in the cytoplasm, the amount of unsaturated fatty acids and total proteins in the plasma membrane, and the content of soluble proteins in the leaf are also increased. Meanwhile, the activities of some detoxification enzymes, such as catalase (CAT), superoxide dismutase (SOD), peroxidase (POD) and esterase (EST) are increased, whereas the metabolic activity is decreased [11,12]. Some cold-induced genes have been cloned in tea plants [13,14]. As a complex biological phenomenon, the ability of tea plants to resist the cold is regulated by a series of genes involved in a complex regulatory network [15]. Using an ‘omics’ research strategy to understand the mechanism of CA in tea plants is the key to improving tea productivity and geographical distribution.

RNA-Seq is a recently developed approach using a massively parallel sequencing strategy to generate transcriptome profiles. It has emerged as a cost-effective approach for high-throughput sequence determination and has unprecedentedly improved the efficiency and speed of gene discovery [16,17]. Digital gene expression (DGE) is a tag-based sequencing approach according to which short tags are generated by endonuclease. The expression level of genes in the sample is measured by counting the number of tags generated from each transcript [18]. This study demonstrates the first attempt to use a combination of RNA-Seq and DGE to study the transcriptome profiles in tea plants and thereby gain a deeper insight into the molecular mechanism of CA. The resulting transcriptome profiles from tea plants not only contributes to the in-depth knowledge of the genes involved in CA but also improves our understanding of plant-environment interactions.

## Results and discussion

### Cold tolerance changes in tea plant during the CA process

**Figure 1:**
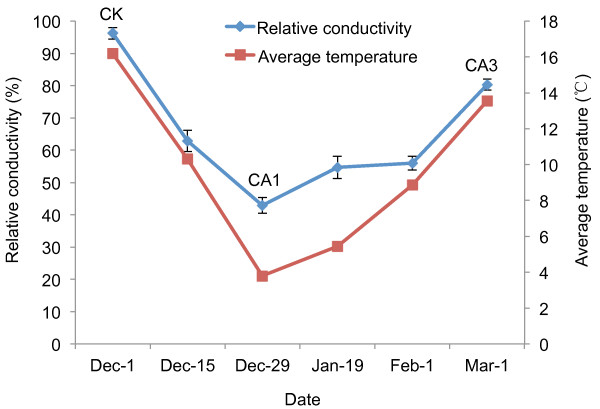
**Changes of relative electrical conductivity and of the averaged air temperature during the CA process of tea plants.** The value of relative electrical conductivity changed from 96.2% for sample CK to 42.9% for sample CA1 when tea plant underwent the cold acclimation process. Then, it returned back to 80.3% (sample CA3) after the air temperature increased. The values of averaged air temperature were also shown during this period.

Cold tolerance in tea plants varies under different temperatures and can be monitored by the relative electrical conductivity using an electrolyte leakage assay. Figure 1 shows a complete course of the CA process for a natural temperature change period from December 2010 to March 2011. Before December 1, the average outdoor temperature (for five days) was above 10°C, and the relative electrical conductivity of tea-plant leaves (CK) was at ~100%, indicating that the tea plant has a low level of cold tolerance. After the tea plant underwent a period of time at relatively low temperatures (<10°C), its relative electrical conductivity decreased, and the cold tolerance of the tea plant is enhanced. When temperatures decreased to their lowest point, the relative electrical conductivity also reached its lowest level with the cold tolerance being at the highest level (CA1). Afterwards, the temperature rose and when the average temperature reached above 10°C, the relative electrical conductivity increased to over 80% and then maintained at a high level. The tea plant was subsequently de-acclimated (CA3), and its cold tolerance was weak (Figure 1).

To obtain the transcriptomic response to the cold environment during the CA process, we selected tea-plant leaves from three stages, non-acclimated (CK), fully acclimated (CA1) and de-acclimated (CA3) for RNA-Seq and digital gene expression (DGE) studies.

### RNA-Seq and *de novo* assembly

We performed RNA-Seq analyses for CA1, CA3 and CK using the Illumina HiSeq2000 genome analyzer. Totally, 57.35 million paired-end reads with a read length of 90 bp were generated (hereafter referred to as dataset 1, Table 1). Since low-quality nucleotides from the ends of reads may lead to incorrect assembly outputs [19], we trimmed the low-quality or ambiguous nucleotides at both ends of the reads. *De novo* assembly was performed with the trimmed reads using Trinity [20]. Trinity was specially developed for *de novo* assembly from short-read RNA-Seq data, which has been shown to be the best single *k*-mer assembler [19,20]. In total, 226,026 transcripts were reconstructed. After removing the redundant transcripts caused by small variations as described in the previous study [19], a final set of 216,831 transcripts were obtained. The average transcript size is 356 bp, and the N50 is 529 bp (Table 2).

**Table 1:** **Summary for RNA-Seq datasets of *C. sinensis***

	**CK**	**CA1**	**CA3**	**Total**
Number of reads (million)	17.54	13.65	26.16	57.35
Total bases (raw, Gb)	1.58	1.23	2.35	5.16
Total bases (trimmed, Gb)	1.50	1.16	2.30	4.96

**Table 2:** **Summary for the outcomes of *de novo *transcriptome assembly using three datasets in *C. sinensis***

	**Dataset 1**	**Dataset 2**	**Dataset 3**
Number of transcripts (≥100 bp)	226,026	177,439	295,551
Number of transcripts (remove redundancy)	216,831	170,650	282,395
Total base pairs (Mbp)	77.36	50.36	94.71
Average length (bp)	356	295	335
N50 (bp)	529	403	480
Mapping ratio (%)	76.94	64.87	76.91

The transcriptome of *C*. *sinensis* was reported in a previous study by Shi et al. [21]. They produced RNA-Seq data from the mixed tissues of *C*. *sinensis* using Illumina GA IIx (hereafter referred to as dataset 2). A combination of dataset 1 and dataset 2 was also generated, which we called dataset 3, representing all available RNA-Seq data for *C*. *sinensis*. Short reads of dataset 2 and dataset 3 were pre-processed by the procedure described above, and then used separately for *de novo* assembly. The assembly outcome from dataset 1 attains the longest average read length and N50, while that from dataset 3 yields the most number of transcripts and total base pairs (Table 2). In order to evaluate the efficiency of short-read usage during the *de novo* assembly, we mapped our RNA-Seq reads back to three sets of reconstructed transcripts, respectively. Transcripts produced from dataset 1 achieved the best performance, with the highest mapping ratio for our short reads (Table 2). More than 10% of the short reads failed to be aligned if only dataset 2 was used for the *de novo* assembly, indicating that previous transcriptome sequences of *C*. *sinensis* are far from saturated. Although more transcriptome sequences could be produced from *de novo* assembly using dataset 3 than dataset 1, the mapping ratio could not be improved (Table 2), indicating that the additional transcripts from dataset 3 are most likely transcripts that are expressed in tissues other than the leaves of tea plants. Thus these additional transcripts are unable to contribute to this study. Based on this scenario, we chose the transcripts from dataset 1 to carry out the downstream analysis.

### Functional annotation of *C*. *sinensis* transcriptome

To predict and analyze the function of the assembled transcripts, non-redundant sequences were submitted to a BLASTx (E-value ≤ 10^-5^) search against the following databases: the NCBI’s NR database, UniRef90 [22], the Arabidopsis Information Resource (TAIR, version 10), Kyoto Encyclopedia of Genes and Genomes (KEGG, version 58) [23] and Clusters of Orthologous Groups from 7 eukaryotic complete genomes (KOG) [24]. We found that about one third of all non-redundant transcripts had significant homology with genes in either the NR or UniRef90 databases (Table 3).

**Table 3:** **Summary for the BLASTx results of *C. sinensis *transcriptome against five databases**

	**Sequences (n)**
**Transcripts**	
Number of transcripts	216,831
BLASTx against NR	716,831
BLASTx against UniRef90	71,709
BLASTx against TAIR10	60,392
BLASTx against KEGG	21,194
BLASTx against KOG	41,341
All annotated transcripts	72,967
Transcripts hit all five databases	16,430
**Unigenes**	
Number of unigenes	179,753
BLASTx against NR	51,883
BLASTx against UniRef90	52,217
BLASTx against TAIR10	42,969
BLASTx against KEGG	15,086
BLASTx against KOG	29,356
All annotated unigenes	53,201
Unigenes hit all five databases	11,388

*Arabidopsis thaliana* is one of the most well-studied dicot plants, with a complete reference genome and comprehensively annotated gene sequences. A BLAST search against genes from *Arabidopsis* produced more definitive annotations and helped us to evaluate the quality and coverage of our assembled transcripts. It is notable that 16,882 *Arabidopsis* genes located uniformly on five chromosomes were covered by 60,392 transcripts (Tables 3 and 4).

**Table 4:** **Distribution of BLASTx hit genes against *Arabidopsis *TAIR10**

**Chromosome**	**Chr1**	**Chr2**	**Chr3**	**Chr4**	**Chr5**	**ChrC**	**ChrM**	**Total**
Total genes	9,263	5,560	6,908	5,356	8,089	88	122	35,386
No. of hits	4,374	2,445	3,293	2,600	4,080	36	54	16,882
%	47.22%	43.97%	47.67%	48.54%	50.44%	40.91%	44.26%	47.71%

A BLAST analysis of the assembled transcripts against the KEGG database showed that 21,194 transcripts were annotated with corresponding Enzyme Commission (EC) numbers and assigned to the reference canonical KEGG pathways (Table 3). A search against the KOG database reported that 41,341 transcripts had the best hits when the E-value was less than or equal to 10^-5^. Since some transcripts could be assigned multiple KOG functions, altogether 46,291 functional annotations were produced and all hit transcripts were grouped in 25 categories (Tables 3 and 5).

**Table 5:** **KOG functional classification of *C. sinensis *transcripts**

**Description**	**Code**	**Transcript (n)**
**1 Information storage and processing**		
Translation, ribosomal structure and biogennesis	J	1,812
RNA processing and modification	A	1,729
Transcription	K	2,341
Replication, recombination and repair	L	1,194
Chromation structure and dynamics	B	706
**2 Cellular processing and signaling**		
Cell cycle control, cell division, chromosome partitioning	D	1,091
Nuclear structute	Y	274
Defense mechanisms	V	385
Signal transduction mechanisms	T	4,836
Cell wall/membrane/envelope biogenesis	M	558
Cell motility	N	24
Cytoskeleton	Z	1,072
Extracellular structure	W	187
Intracellular trafficking, secretion, and vesicular transport	U	2,289
Posttranslational modification, protein turnover, chaperones	O	4,152
**3 Metabolism**		
Energy production and conversion	C	1,800
Carbohydrate transport and metabolism	G	2,387
Amino acid transport and metabolism	E	1,514
Nucleotide transport and metabolism	F	432
Coenzyme ransport and metabolism	H	343
Lipid ransport and metabolism	I	1,440
Inorganic ion ransport and metabolism	P	1,159
Secondary metabolites biosynthesis, transport and catabolism	Q	1,428
**4 Poorly characterized**		
General function prediction only	R	11,108
Function unknown	S	2,030

**Figure 2:**
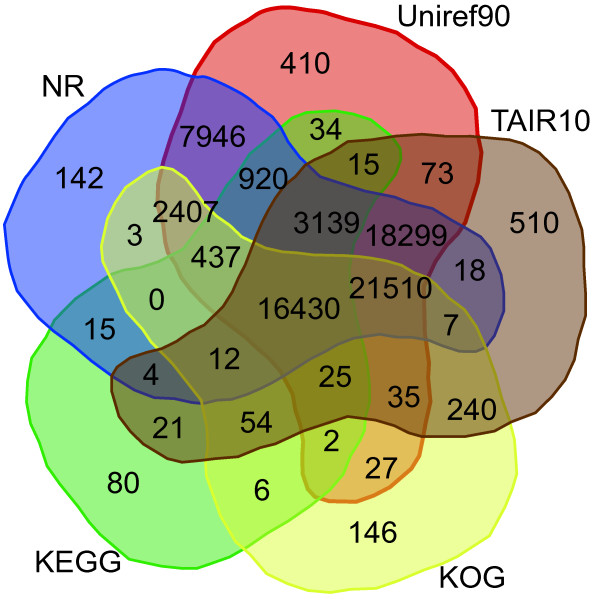
**Venn diagram showing the BLAST results of *C. sinensis *transcriptome against five databases. ***De novo* reconstructed transcript sequences were used to BLAST search against public databases including NCBI’s NR, UniRef90, TAIR10, KEGG and KOG. The number of transcripts that have significant hits (E-value ≤ 10^–5^) against the five databases is shown in each intersection of the Venn diagram.

In total, 72,967 transcripts got the best hits with known proteins in at least one of the five databases and 16,430 transcripts had similarity to proteins in all of the five databases (Figure 2, Table 3).

**Figure 3:**
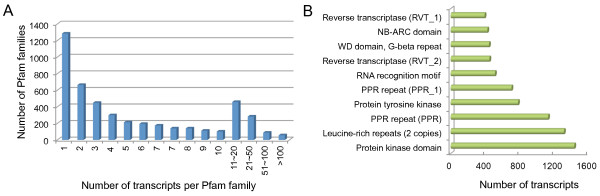
**Protein families in *C. sinensis *transcriptome.** Shown are the number of Pfam domains/families versus the occurrence of *C*. *sinensis* transcripts contained in each domain/family (**A**), and the 10 most abundant protein families (**B**) in *C*. *sinensis*.

To functionally categorize the assembled transcripts, gene ontology (GO) [24] terms were assigned to each transcript based on the best BLASTx hit from the NR database using Blast2GO [25]. Out of 71,289 transcripts with NR annotation, 30,115 transcripts were assigned 80,176 GO term annotations in three main GO categories including biological process, cellular component and molecular function (Table 3, Additional file 1). If a gene contained some conserved domains, the domain information would be useful for interpreting the gene’s function. To annotate the potential domains inside the reconstructed sequences, the open reading frame (ORF) was predicted for each transcript (Methods), and then all transcripts with predicted ORF were used to search against the Pfam database based on profile hidden Markov model methods. In total, 41,599 transcripts were assigned Pfam domain information and were categorized into 4,504 domains/families. Most domains/families were found to contain a small number of transcripts (Figure 3A). According to the frequency of the occurrence of *C*. *sinensis* transcripts contained in each Pfam domain, Pfam domains/families were ranked and the top 10 abundant domains/families are listed in Figure 3B, with hit results similar to the previous study [21]. Among these domains/families, “Protein kinase domain” and its subclass “Protein tyrosine kinase” are known to regulate the majority of cellular pathways. Proteins with “leucine-rich repeats” domain are known to be frequently involved in the formation of protein–protein interactions, and “PPR repeat” has been reported to be a large protein family in plants with versatile functions [26]. Moreover, the “NB-ARC” protein family, comprised of resistance proteins, was highly represented. Other protein families, such as “reverse transcriptase” and “RNA recognition motif”, which have some basic functions in plants, were also found in the top ten of the list.

Trinity produced all potential alternative spliced isoforms during the *de novo* assembly, and isoforms originated from the same gene locus were assumed to share the same “chrysalis component”, “butterfly sub-component” and some of the paths in the *de Bruijn* graph. We chose the longest transcript in each locus to get the unigene set, resulting in 179,753 unigenes (Table 3). Potential isoforms in each locus reported by Trinity would be useful in array/primer design for quantitative gene expression and future alternative splicing analyses.

### Identification of genes involved in cold acclimation

The abundance estimation for reconstructed transcripts was determined by RSEM software package that was shown to have the ability to effectively use ambiguously-mapping reads and to accurately estimate isoform-level abundance for *de novo* assembled transcripts without reference [27]. The DESeq package [28] and the winflat program were then applied to identify differentially expressed genes. CA-related genes were identified based on the fold change on the abundance of each gene and the corresponding false discovery rate, which resulted in 1,770 differentially expressed genes. Of these, 1,168 were up-regulated and 602 were down-regulated (Additional file 2), indicating that more genes were activated than repressed during the CA process. Dozens of cold-regulated or cold-related genes were found in this differential expression list, including cold sensor or signal transduction genes, cold-responsive transcription factor genes, plasma membrane stabilization related genes, osmosensing-responsive genes and detoxification enzymes genes.

### Cold sensor or signal transduction genes

The signal transduction pathway plays a pivotal role in the response to the stress of low temperatures [29]. It is well known that Ca^2+^ acts as a key messenger in regulating growth and developmental processes and plays a crucial role in stress signaling, i.e. cold stress [30]. Cold stress could activate Ca^2+^ channels to increase the cytosolic Ca^2+^ level, and then trigger phospholipase C and D, producing inositol triphosphate and phosphatidic acid, respectively. Inositol triphosphate could further amplify Ca^2+^ signatures, and phosphatidic acid is proposed as a membrane-based secondary messenger molecule [31,32]. Subsequently, many signaling pathways are triggered, such as Ca^2+^-dependent protein kinases (CDPKs), mitogen-activated protein kinases (MAPKs), calcineurin B-like protein (CBL), calmodulin, etc. Doherty et al. [33] and Yang et al. [34] found that the calmodulin-binding transcription activator (CAMTA) and a novel calcium/calmodulin-regulated receptor kinase (CRLK1) were crucial for cold tolerance in plants. Ca^2+^ influx into the cell was considered to occur upstream of the expression of CBFs and COR genes in the cold signaling pathway [35,36]. In this study, 13 genes, which were annotated as CDPKs, CBL, calmodulin, CAMTA, MAPK and phospholipase, were identified as being involved in signal transduction upon low temperature stress. Among these genes, 9 (4 calmodulin genes, 2 CDPK genes, 1 CAMTA gene and 2 phospholipase genes) were up-regulated in CA1, whereas 4 (1 phospholipase gene, 1 calmodulin gene, 1 CBL gene and 1 MAPK gene) were down-regulated.

Plant protein kinases belong to a large superfamily, some of which have been known to play a central role in cellular signaling, for example CDPKs and MAPKs. In addition, a growing body of evidence has shown that receptor-like kinases (RLKs) are involved in the perception of environmental signals [37,38]. Histidine kinases (HKs), being localized to the cellular membranes and endoplasmic reticulum, are the major signaling molecules and are involved in the two-component signaling pathways that mediate plant-sensed environmental signals and regulate the downstream environmental stress response [39,40]. In this study, 27 RLKs genes and 2 HKs genes were differentially expressed and all of these were up-regulated in CA1 samples, which indicates that protein kinases play an important role in the CA process in tea plants.

### Cold-responsive transcription factor genes

Transcription factors (TFs) play important functions in plant development and stress tolerance [35]. Fifty-eight genes encoding putative TFs in *C*. *sinensis* were identified. These TFs could be divided into 9 groups (AP2/ERF, bHLH, WRKY, MYB, NAC, bZIP, heat shock, GARS and zinc finger protein) based on the classification of their *Arabidopsis* homologs, and most of them have been reported to be linked to cold stress resistance in plants [41-49]. Among these TFs, 37 genes were up-regulated and 21 genes were down-regulated in our CA1 sample.

Of the 9 groups of TFs, zinc finger was the most enriched TF family, containing 31 genes of the 58 cold-responsive TFs, with 18 genes being up-regulated and 13 being down-regulated. There were 5 genes in the bHLH family (4 up- and 1 down-regulated), 5 genes in the MYB family (4 up- and 1 down-regulated), 5 genes in the WRKY family (3 up- and 2 down-regulated) and 3 genes in the NAC family (1 up- and 2 down-regulated). In addition, 2 genes in the bZIP family, 3 genes in the GARS family and 2 genes encoding heat shock proteins were all up-regulated, while 2 genes in the AP2/ERF family were down-regulated in the CA1 sample. It is interesting to find down-regulated genes in the AP2/ERF family, as these suggest that the interaction of light and temperature is of special importance for plants during the CA process. Catalá et al. [50] and Jurczyk et al. [51] have also reported that light is required for full CA in *Arabidopsis* and *Festuca pratensis*.

### Genes related to the stabilization of the plasma membrane and osmosensing-responsiveness

The plasma membrane is believed to be a primary site of injury from freezing in plants. The process of CA can stabilize the membrane structure and prevent it from damage [52]. Under freezing temperatures, membranes must be kept fluid in order to sustain the functional activity of membrane proteins and membranes themselves [53]. Alterations occur in the composition of proteins and lipids (e.g. increases in the unsaturation level of the membrane lipids) in the plasma membrane in response to CA, and these are associated with an increase in freezing tolerance [53,54]. In our study, we identified 3 lipid-transfer protein (LTP) genes and 1 fatty acid desaturase (FAD) gene. Among these, 2 LTP genes and a FAD gene were up-regulated and 1 LTP gene was down-regulated. These genes were known to regulate the level of unsaturated fatty acids, and then to further mediate the regulation of membrane fluidity [55-57].

Moreover, in order to maintain the structural stabilization of the plasma membrane during the CA process, some proteins function as inhibitors to regulate the activity of ice nucleators. These proteins are so-called anti-freezing proteins (AFPs), such as β-1, 3-glucanase-like proteins (GLPs), chitinase-like proteins (CLPs), thaumatin-like proteins (TLPs), polygalacturonase inhibitor proteins (PGIPs) and late-embryogenesis-abundant proteins (LEAs). In the CA1 sample, more genes encoding these proteins were up-regulated compared with genes in non-acclimated samples [36,58]. In our study, we found 7 AFP-related genes, including 4 CLPs, 1 TLP, 1 PGIP and 1 LEA that were up-regulated in the CA1 sample, indicating that during the CA process, tea plants became able to tolerate freezing temperatures through the enhancement of membrane stability.

The stabilization of the plasma membranes is also related to the osmotic equilibrium. In order to maintain osmotic balance, plants accumulate a range of compatible solutes, including soluble sugars (saccharose, trehalose, rafinose), sugar alcohols (ribitol, inositol, sorbitol), and low-molecular-weight compounds (such as proline, glycine betaine, glutamic acid) as cryoprotectant molecules in response to cold stress [59-61]. Accordingly, the expression of these metabolism related genes also changes during CA [29,36,62]. We identified 13 genes related to the carbohydrate metabolic pathway from 1,770 differentially expressed genes, including 4 galactosidases (3 up- and 1 down-regulated), 5 amylases (all up-regulated), 1 galactinol synthase (up-regulated), 1 raffinose synthase (up-regulated) and 2 trehalose-6-phosphate synthases (all up-regulated). These genes are key genes of the carbohydrate metabolic pathway, and are closely involved with the CA process [63]. Three monosaccharide transporter genes (2 up- and 1 down-regulated) were identified as well. Monosaccharide transporters play an important role in sugar transport and distribution in plants. The expression of monosaccharide transporter genes is also regulated by cold stress [64]. These results suggested that the carbohydrate metabolic pathway plays a critical role in tea plants during the CA process.

### Validation of RNA-Seq results by DGE and qRT-PCR

Digital gene expression (DGE) library sequencing was performed to validate the cold-regulated transcripts identified by RNA-Seq. In our study, three DGE libraries were sequenced: CA1, CA3 and CK, for which 3.69, 3.62 and 3.68 million raw tags were generated, respectively (Table 6). After removing low-quality tags, the total number of clean tags per library ranged from 3.53 to 3.60 million. Clean tags from three DGE libraries were mapped onto our assembled transcriptome sequences. Up to 24.25% of transcripts were detected by DGE tags (Table 6).

**Table 6:** Summary for DGE datasets

**Summary**	**CA1**	**CA3**	**CK**
Raw tags	3,689,000	3,615,500	3,675,000
Clean tags (total)	3,599,673	3,528,122	3,586,229
Aligned tags	2,027,613	2,402,599	1,810,760
Aligned tags (%)	56.33%	68.10%	50.49%
Clean Tags (unique)	882,038	456,976	932,551
Aligned unique-tags	117,591	104,604	111,505
Aligned unique-tags (%)	13.33%	22.89%	11.96%
Transcripts with aligned tags	52,590	46,331	51,333
Transcripts with aligned tags (%)	24.25%	21.37%	23.67%
Transcripts with uniquely aligned tags	33,380	28,462	32,519
Transcripts with uniquely aligned tags (%)	15.39%	13.13%	15.00%

**Figure 4:**
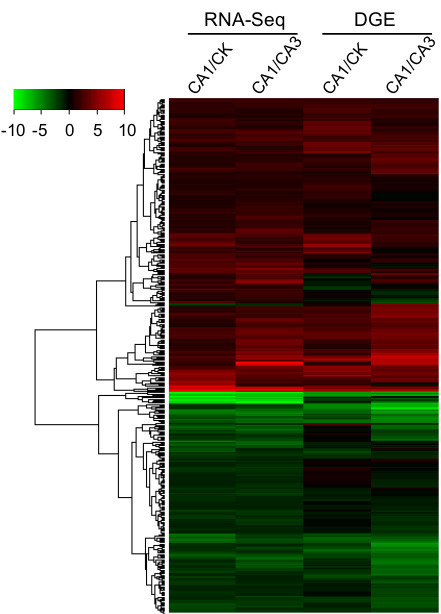
**Heatmap representing relative expression levels of 398 differentially expressed transcripts in tea plants*. ***Expression levels were determined by RNA-Seq and DGE, respectively. Cluster analysis of differentially regulated transcripts expressed in CA1 versus CA and CA3 samples indicating the high degree of consistency between DGE and RNA-Seq platforms.

Of the 1,770 differentially expressed transcripts identified by RNA-Seq, 1,460 were detected by DGE sequencing, but 870 were mapped by uncertain tags (tags that mapped to at least two different transcripts) and another 192 transcripts did not have enough tags (< 1 ‘tags per million’ (TPM) counts for all three samples) to differentiate expressions among CA1, CA3 and CK samples. This result illustrates that DGE sequencing was limited to identify differential expression across the full scale of transcriptome profiles, especially for genes with paralogs or multiple isoforms that shared the same tags (CATG+17bp). Of the remaining 398 transcripts, the majority of them (376/398) showed consistent expression patterns between DGE and RNA-Seq, with the corresponding Pearson’s r being 0.77 and 0.81 for CA1/CA3 and CA1/CK, respectively (Figure 4, Additional file 3), demonstrating the degree of consistency between DGE and RNA-Seq platforms.

It is worth noting that some transcripts, though not many, showed different expression patterns in the profiling results from RNA-Seq and DGE (Figure 4). Determining which method is more robust and why the two approaches yield different results would be useful for identifying the correct outcomes in this study and for other researchers to choose the appropriate approach in their future studies. To address this, 10 of these transcripts that showed inconsistent results from RNA-Seq and DGE platforms were randomly selected to assess their relative expression patterns among CK, CA1 and CA3 using quantitative RT-PCR approach (qRT-PCT). For most of these (8/10), similar expression patterns were observed compared with those from RNA-Seq results, while in the other 2 transcripts there were only partial consistencies with either RNA-Seq or DGE results (Additional file 4). In general, RNA-Seq outperforms DGE based on the results from these 10 cases. The less accurate estimation of the gene expression level by DGE approach could be due to some unknown reason(s) or to the fact that the same tags may exist in other transcripts that were partially reconstructed after *de novo* transcriptome assembly and lack the complete tag sequences. Since the DGE approach counts all tags to the transcript with the exactly matched tag sequences, this may result in the incorrect estimation of the expression level for some transcripts. In the remaining two genes, inconsistent expression patterns were observed among the results from the three approaches. These genes expressed at relatively variable levels may be affected by factors other than a cold environment and these kinds of false positives could be largely avoided if more biological replicates were included.

The DGE method is widely applied for studying the transcriptome. However, it has limitations in presenting a global view of transcriptome profiles. It is powerless to detect the abundance of transcripts when 1) there is no CATG site in transcripts or 2) several transcripts share the same tag, situations involving two unrelated genes, paralogs, or alternatively spliced isoforms. Both closely related paralogs and alternatively spliced isoforms might exhibit various spatial and temporal expression patterns, or even have different functions. Thus, the ability to correctly estimate isoform expression levels will be necessary for understanding complicated biological mechanisms.

**Figure 5:**
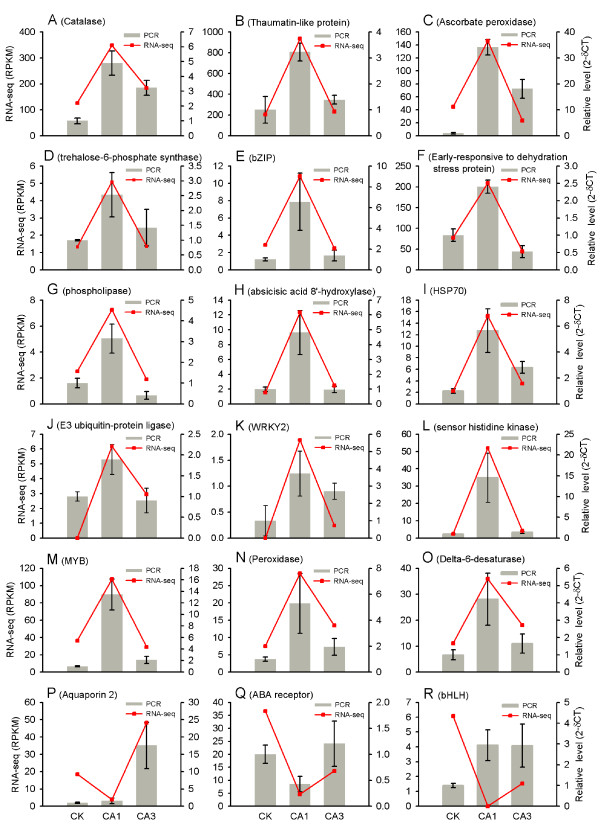
**Quantitative RT-PCR validations.** 18 genes were selected for the quantitative RT-PCR analysis, including catalase (**A**), thaumatin-like protein (**B**), ascorbate peroxidase (**C**), trehalose-6-phosphate synthase (**D**), bZIP (**E**), early-responsive to dehydration stress protein (**F**), phospholipase (**G**), absicisic acid 8′-hydroxylase (**H**), HSP70 (**I**), E3 ubiquitin-protein ligase (**J**), WRKY2 (**K**), sensor histidine kinase (**L**), MYB (**M**), peroxidase (**N**), delta-6-desaturase (**O**), Aquaporin 2 (**P**), ABA receptor (**Q**) and bHLH (**R**). The18S ribosomal gene was chosen as the reference gene.

To further test the reliability of outcomes produced from the next generation sequencing platform, quantitative RT-PCR (qRT-PCR) analysis was performed for 18 of the 1,770 differentially expressed transcripts. These 18 transcripts were manually selected as representatives for their potential roles in cold tolerance according to their annotations. The expression patterns of 17 genes detected by qRT-PCR fit well with those from RNA-Seq results, with one annotated as a basic helix-loop-helix DNA-binding superfamily protein being inconsistent (Figure 5).

### Pathways involved during the CA process in *C*. *sinensis*

The 1,770 transcripts were used to search the KEGG pathway to determine whether the genes involved in CA were from specific pathways. In total, 200 pathways were identified, 20 of which were significantly enriched during CA (*P* < 0.05, Additional file 5). Of these significantly enriched, metabolism was the largest category (99 transcripts), including carbohydrate metabolism (28), glycan biosynthesis and metabolism (21), energy metabolism (15), amino acid metabolism (14), metabolism of terpenoids and polyketides (9), enzyme families (5), xenobiotics biodegradation and metabolism (4) and lipid metabolism (2). Moreover, calcium signaling pathway (9) and membrane transport pathway (7) were enriched as well. Numerous studies reported that carbohydrate metabolism plays an important role during the CA process [65-69]. In this study, metabolic pathways for carbohydrates stood out from the enrichment analysis, including pathways for 49 differentially expressed transcripts (28 for carbohydrate metabolism and 21 for glycan biosynthesis and metabolism), indicating that the regulation of carbohydrates is crucial for tea plants during CA.

Previous studies have shown that calcium acts as a pivotal mediator in the signal transduction pathway during the CA process [29,36]. Calcium is a secondary messenger in plant signaling processes, and calcium/calmodulin-mediated signaling is believed to play an important role in plants during the cold stress response [30,34,70]. In this study, the calcium signaling pathway was also enriched, and most of the genes in this pathway were up-regulated in the CA1 sample, proving the importance of the calcium signaling pathway for the tea plants’ response to cold stress.

## Conclusions

**Figure 6:**
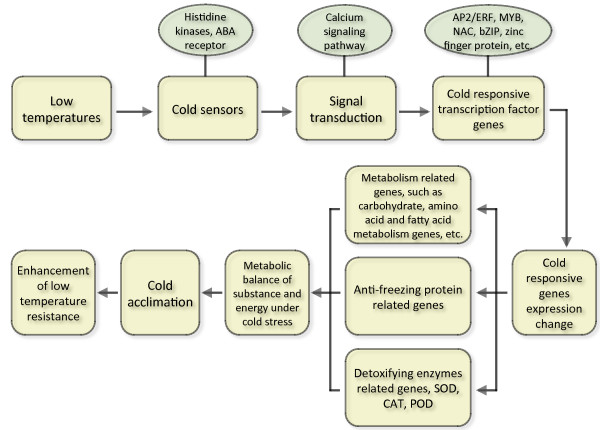
A diagram of tea plants’ responses to low temperatures during the CA process.

In this study, we present a global survey for transcriptome profiles in tea plants during the CA process using RNA-Seq and DGE. A large number of genes from tea plants involved in diverse biological or molecular pathways were identified during the CA process, such as genes involved in cold signal sensors or transduction, genes related to the stabilization of plasma membranes, osmosensing-responsive genes, and stress-responsive transcription factor genes. A diagram is shown to illustrate tea plants’ responses to low temperatures during the CA process (Figure 6). The results showed that a series of complex regulatory networks were triggered in tea plants during CA. Our study provides insights into the molecular mechanisms of tea plants during the CA process. It could also serve as a valuable resource for relevant research on cold-tolerance and help to explore the cold-related genes in improving the understanding of low-temperature tolerance and plant-environment interactions.

## Methods

### Low-temperature tolerance assays and RNA preparation

The tea plant cultivar ‘*Camellia sinensis* (L.) O. Kuntze cv. *Longjing 43*’ was planted in the China National Germplasm Hangzhou Tea Repository (CNGHTR) at the Tea Research Institute, Chinese Academy of Agricultural Sciences (TRI, CAAS). Starting in October 2010, intact mature leaves were selected in every 10–15 days until March 2011, when the average temperature became higher than 15°C. All samples were washed with distilled deionized water and divided into two parts, one for −80°C storage using liquid nitrogen for quick freezing and the other for evaluating low-temperature tolerance using an electrolyte leakage assay. RNAprep pure Plant Kit (Tiangen, Beijing, China) was used for total RNA extraction, and Agilent 2100 Bioanalyzer was used to test the RNA integrity with a minimum integrity value of 8.

The low-temperature tolerance was determined from leaf samples by electrolyte leakage assay similar with previous study [71]. Briefly, leaves were washed with deionized water. Leaf samples (5 mm in diameter) were extracted using a hole puncher and the midvein of the leaf was excluded. Leaf samples (0.5 g) were placed in closed vials containing 20 ml of deionized water and incubated at 25°C on a rotary shaker for 24 h. Then the electrical conductivity of the solution (L1) was determined. Samples were then autoclaved at 100°C for 20 min and the final electrical conductivity (L2) was determined after equilibration at 25°C. The EL (relative electrical conductivity) was defined as follows: EL (%) = (L1/L2×100%). Based on the level of electrolyte leakage, three samples including non-acclimated (CK, the date for sample collection was 1st December, 2010), fully acclimated (CA1, the date was 29th December, 2010) and de-acclimated (CA3, the date was 1st March, 2011) were selected for RNA-Seq and DGE analyses.

### Library preparation and RNA-Seq

The samples for RNA-Seq were prepared using Illumina’s kit and following manufacturer’s recommendations. In short, mRNA was purified from 20 μg of total RNA using oligo (dT) magnetic beads, followed by fragmentation, in which the mRNA is fragmented into small pieces using divalent cations under elevated temperature. The cleaved RNA fragments were used for first-strand cDNA synthesis using reverse-transcriptase and random primers followed by second-strand cDNA synthesis using DNA polymerase I and RNase H. After the end repair process and ligation of adapters, the products were enriched by PCR to create the final cDNA library.

The cDNA library was sequenced from both 5′ and 3′ ends using the Illumina HiSeq™ 2000 platform according to the manufacturer’s instructions. The fluorescent image processing, base-calling and quality value calculation were performed by the Illumina data processing pipeline 1.4, in which 290 bp paired-end reads were obtained.

### Short-read RNA-Seq datasets

In our study, we performed RNA-Seq for three samples from tea plants that represented three key stages during the CA process, including CA1, CA3 and CK. We called these dataset 1. The accession code of our RNA-Seq dataset is SRA061043. The previous study reported the transcriptome of *C*. *sinensis*, with 75 bp paired-end reads produced from the Illumina GAII platform, and we called this dataset 2. Its accession code is SRX020193, which includes samples from seven different tissues of *C*. *sinensis*: tender shoots, young leaves, mature leaves, stems, young roots, flower buds and immature seeds [21]. Furthermore, we combined dataset 1 and dataset 2 together as dataset 3 in order to compare the outcomes from *de novo* assembly using different datasets.

### Preprocessing and *de novo* assembly

Raw data is preprocessed before *de novo* assembly: low-quality nucleotides (we defined nucleotides with a quality score less than 20 as low-quality nucleotides) in the last 20 cycles and ambiguous nucleotides in the first five cycles were trimmed by custom PERL script. After preprocessing, we obtained a total of ~ 4.96 G bases (Gb), ~ 1.90 Gb and ~ 6.86 Gb quality filtered short reads for dataset 1, dataset 2 and dataset 3, respectively.

*De novo* assemblies for these three datasets were performed separately by Trinity (release 20110713) [20]. The command-line parameters are “--seqType fq --left 1.fq --right 2.fq --paired_fragment_length 300 --min_contig_length 100 --run_butterfly --output RNASeq_Trinity --CPU 8”.

### Removal of redundancy

Some isoforms reconstructed by Trinity with the same “chrysalis component” and “butterfly sub-component” had only small variations, such as SNPs, small insertions or deletions; such variations introduced redundancies for the assembly outcomes. CD-HIT-EST [72] was used to remove the shorter redundant transcripts when they were entirely covered by other transcripts with more than 99% identity. This set of transcripts was then used to count the basic assembly statistics and for downstream analysis.

### Gene annotation and classification

All non-redundant transcripts (≥ 100 bp) were used to search against the NR, UniRef90 [22], TAIR10, KEGG (version 58) [23] and KOG [24] databases by BLASTALL package (release 2.2.22) with the significant threshold of E-value ≤ 10^-5^. Each known gene from the best BLASTx hit was parsed and assigned. Gene ontology (GO) [73] terms for each transcript were assigned based on the best BLASTx hit from the NR database using Blast2GO software (version 2.3.5) [25] with an E-value threshold of 10^-5^.

The ORF of assembled transcripts was determined based on the results of BLASTx search in the following order: NR, UniRef90, KEGG and KOG. Extending from both sides of the aligned region, the coding region sequences were translated into amino acid sequences with the standard codon table using custom PERL scripts. For those transcripts without any BLASTx hit against known databases, the best potential coding region was predicted using the software BestORF with parameters trained on *Arabidopsis* ESTs. The predicted amino sequences were submitted to search against the Pfam database (version 25.0) [74] for domain/family annotation using HMMER 3.0, with the ‘Best Match Cascade’ protocol. The “optimising allowed match overlap” method [75] was used to resolve complex overlapping protein domains.

### Mapping reads to transcripts

In order to get assembly statistics for the ratio of number of reads that could be mapped back to transcripts (mapping ratio), bowtie (version 0.12.7) [76] was used to align short reads to the reconstructed transcripts, with parameters “-q --solexa1.3-quals --fr −1 fq1 -2 fq2 -k 1 -v 3 -X 300”. Custom PERL scripts were used to summarize the aligned results.

### Calculation of gene expression level

RSEM (v1.1.11) [27] was used to quantify transcript abundance in each sample, with parameters “--phred64-quals --estimate-rspd --calc-ci --out-bam --fragment-length-min 100 --fragment-length-max 350” , and then the RSEM-estimated fragment counts were fed into DESeq package (1.0.6) [28] to get the ‘baseMean’ value. The false discovery rate (FDR) of each comparison (CA1 vs. CA3 and CA1 vs. CK) was calculated by the winflat program which implements a rigorous statistical analysis described by Audic and Claverie [18]. The FDR ≤ 0.01 and the absolute value of log2 ratio ≥ 1 were used as the threshold of significant differences in gene expression. Those genes that were significantly differentially expressed in both CA1 vs. CK and CA1 vs. CA3 were identified as potentially related to CA.

### Digital gene expression

Tag library preparation for three samples was performed in parallel using the Illumina gene expression sample preparation kit. Briefly, 6 μg total RNA from each sample was used for mRNA capture with magnetic oligo (dT) beads. First- and second-strand cDNA were synthesized. Bead-bound cDNA was subsequently digested with *Nla*III. The cDNA fragments with 3' ends were then purified with magnetic beads, and the Illumina adapter 1 was ligated to their 5' ends. The junction of the Illumina adapter 1 and CATG site is the recognition site of MmeI, which cuts the cDNA at 17 bp downstream of the CATG site, producing tags linked with adapter 1. After removing 3' fragments with magnetic beads precipitation, the Illumina adaptor 2 was ligated to the 3' ends of tags. The ligation products were enriched by PCR amplification (15 cycles) and purified by 6% TBE PAGE Gel electrophoresis. Sequencing was carried out on the Illumina HiSeq™ 2000 platform, as recommended by the manufacturer, for 35 cycles.

Raw image data was transformed by base calling into sequence data. Adaptor sequences were removed by custom PERL scripts and low-quality tags with ambiguous nucleotide(s) were discarded. All remaining tags were then aligned to the reconstructed transcripts by bowtie with parameters “-a -f -v 0”. Tags that could not be uniquely aligned were discarded. For gene expression analysis, the number of expressed tags was counted and then normalized to TPM.

### Quantitative real-time RT-PCR (qRT-PCR) analysis

In order to validate the reliability of RNA-Seq and DGE experiments, 28 transcripts were selected for quantitative RT-PCR (qRT-PCR) test. The RNA (1 μg) of each sample was treated with *DNas*e I (Tiangen, China), then real-time PCR was performed using PrimeScript^TM^ RT reagent qPCR Kit fromTakara (Dalian, China) under the following parameters: 95°C for 30 s, 40 cycles at 94°C for 15 s, 60°C for 34 s. Fluorescence intensity was measured using the Applied Biosystems 7300 Sequence Detection System (Carlsbad, CA, USA). Triplicates of each reaction were performed. To ensure the robustness of the reference gene used in the qRT-PCR experiment, we analyzed the gene expression stability of 4 commonly used housekeeping genes (18S RNA, β-Actin, GAPDH and α-Tubulin) across the cold acclimation process. As previously reported by others [77], our results also showed that the 18S RNA gene was the most stable one for its constant expression levels and was finally chosen as the reference gene in our study. The relative expression of the genes in the three samples was calculated using the 2^−ΔΔCt^ method described earlier [78]. The result of the qRT-PCR was presented as fold changes in gene expression relative to that of CK sample. So, the relative value of CK is 1 and the relative values of CA1 and CA3 samples were normalized to that of CK sample. All data are shown as the mean ± SD and all primer information is provided in Additional file 6.

## Competing interests

The authors declare that they have no competing interests.

## Authors’ contributions

YJY, XCW, QYZ and XL conceived and designed the experimental plan. XCW, CLM, HLC, CY, XYH, LC, JQM and JQJ participated in sample collection and performed experiments. QYZ, XCW, ZHZ and YMK performed bioinformatics analyses. XCW and QYZ deposited the sequencing data in the GenBank and SRA databases. XCW, QYZ, XL and YJY drafted and revised the manuscript. All authors read and approved the final manuscript.

## Supplementary Material

Additional file 1 **Gene Ontology classification of *C*. *sinensis *transcriptome.** Gene Ontology (GO) terms are summarized in three main categories: cellular component, molecular function and biological process. The left and right y-axes are in log(10) scale, indicating the percentage and the number of genes within a specific GO category, respectively. Click here for file

Additional file 2 **List of differentially expressed transcripts of *C*. *sinensis *during CA.** Listed are transcript ID (column A), the length of each transcript (B), numbers of raw reads for each transcript (C-E), RPKM (F-H), “baseMean” (I-K), fold change, *P*-value and FDR for CA3/CA1 (L-P) and CK/CA1 (Q-U), and annotations search against NR, UniRef90, TAIR10, KEGG and KOG (V-AH). “baseMean” values were generated by DESeq package. RPKM: reads per kilobase per million mapped reads. Click here for file

Additional file 3 **The relative gene expression values of 398 transcripts based on RNA-Seq and DGE platforms.** Transcript ID (column A), relative gene expression values (log2 ratio) for CA1/CK and CA1/CA3 generated from both DGE (B-C) and RNA-Seq (D-E) are shown. Click here for file

Additional file 4 **Quantitative RT-PCR validations for 10 randomly selected transcripts.** The quantitative RT-PCR experiment was performed on 10 randomly selected transcripts that show distinct results from RNA-Seq and DGE approaches. Consistent expression patterns between RNA-Seq and qRT-PCR results were observed in eight of these transcripts (A, B, C, D, E, H, I and J), while the other 2 transcripts were only partially consistent (F and G). Click here for file

Additional file 5 **Pathways identified in *C*. *sinensis *during CA.** KEGG ko terms (column A), the number of differentially expressed genes in each ko category (B), the number of genes in each ko category (C), *P* value (D), annotations (E-G) and gene ID (H) are shown. Click here for file

Additional file 6 List of designed primers for quantitative RT-PCR. Click here for file
