# Supplementary figures and images for: Global transcriptome profiles of Camellia sinensis during cold acclimation
Source: BMC Genomics. 2013 Jun 22;14:415. doi: 10.1186/1471-2164-14-415 (PMC3701547; doi:10.1186/1471-2164-14-415)

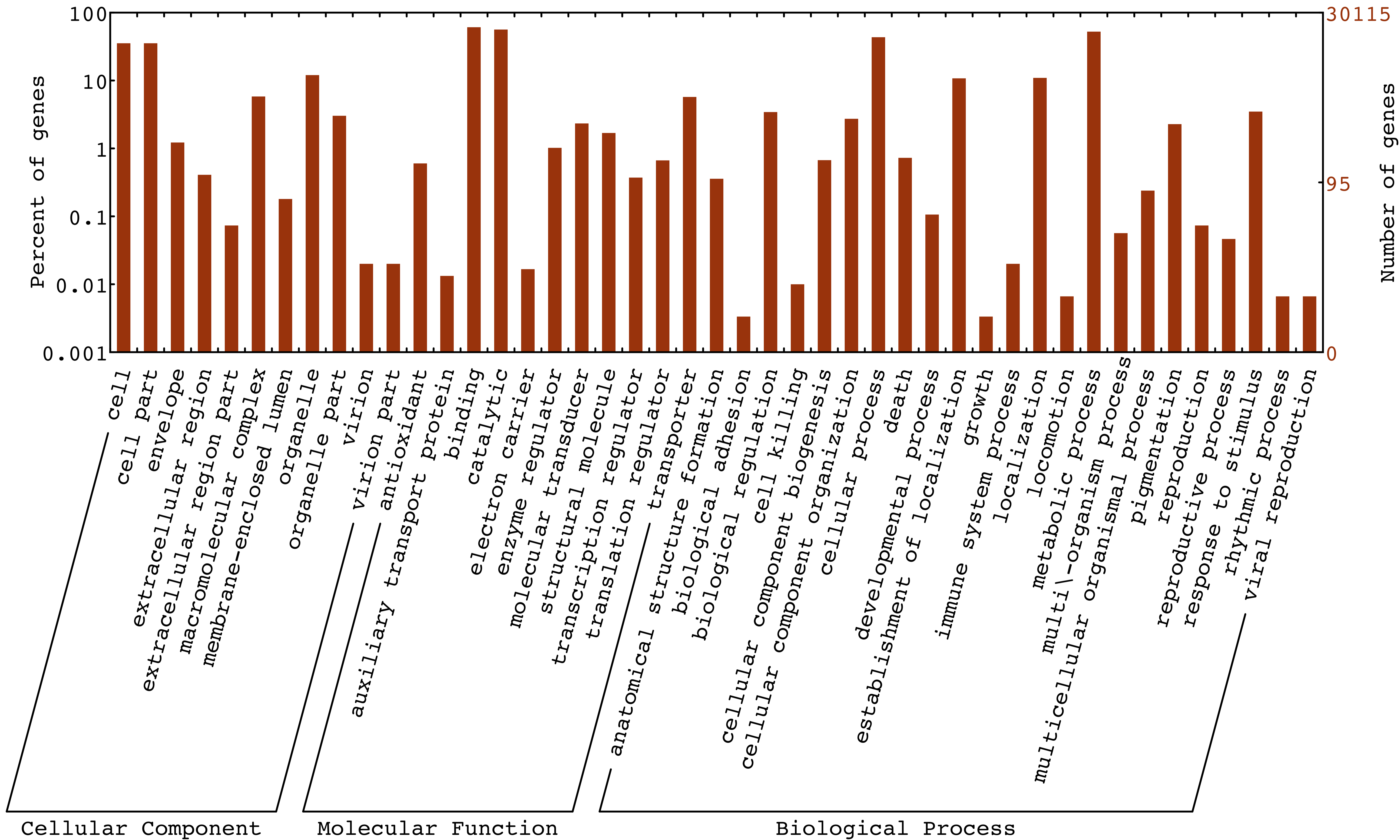

Supplement: Additional file 1 — Gene Ontology classification of C. sinensis transcriptome. Gene Ontology (GO) terms are summarized in three main categories: cellular component, molecular function and biological process. The left and right y-axes are in log(10) scale, indicating the percentage and the number of genes within a specific GO category, respectively. [file 1471-2164-14-415-S1.pdf]

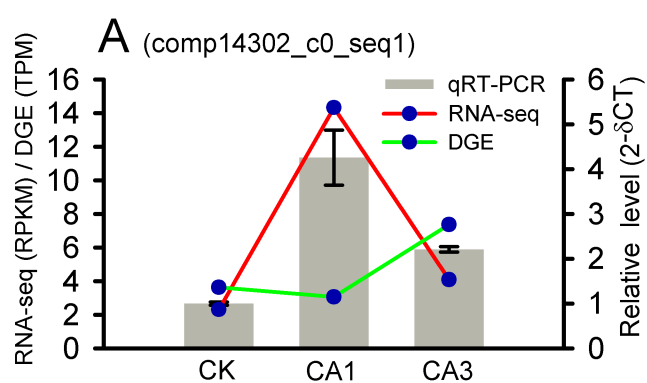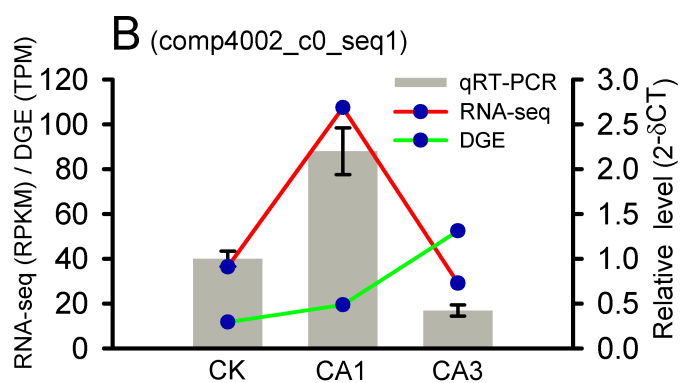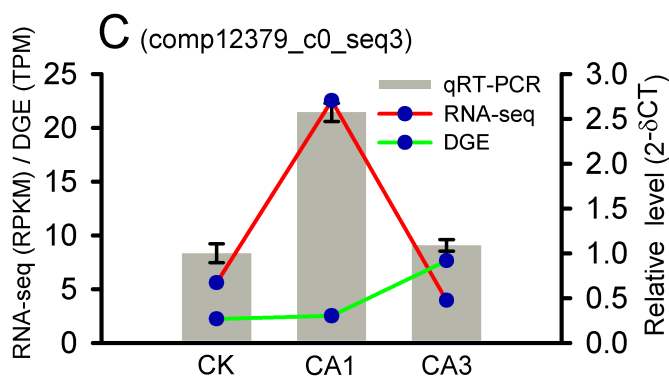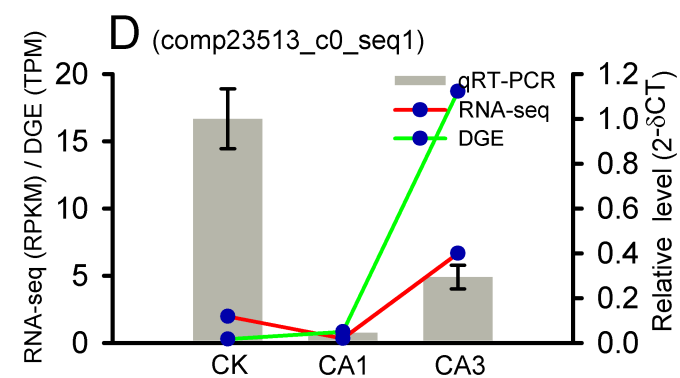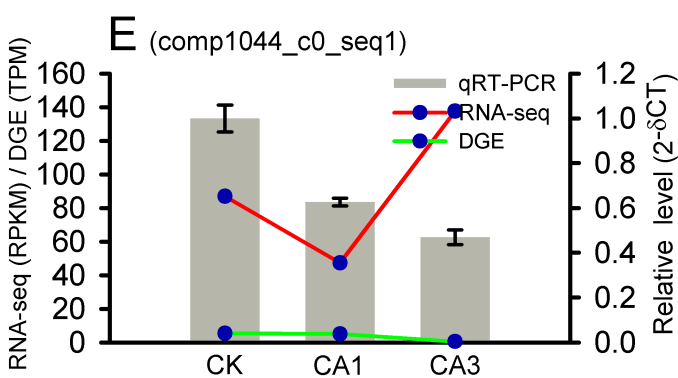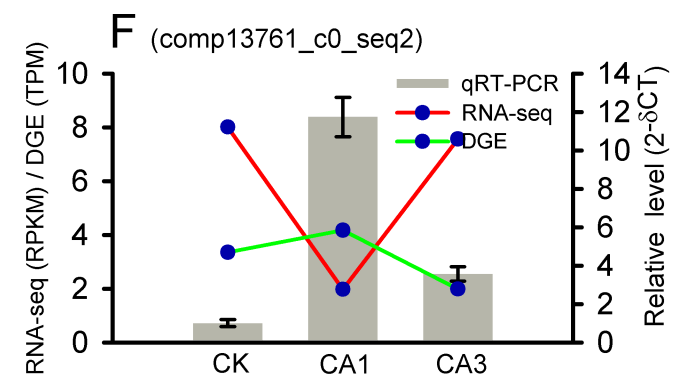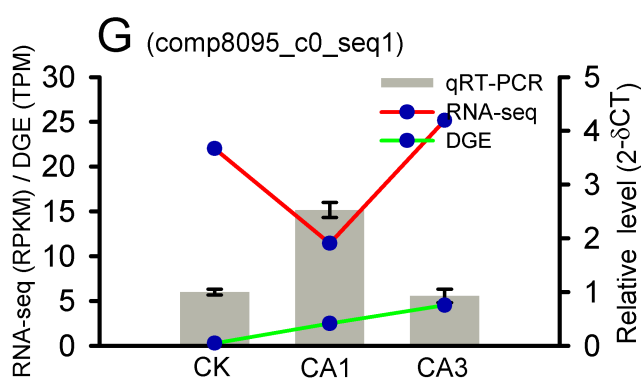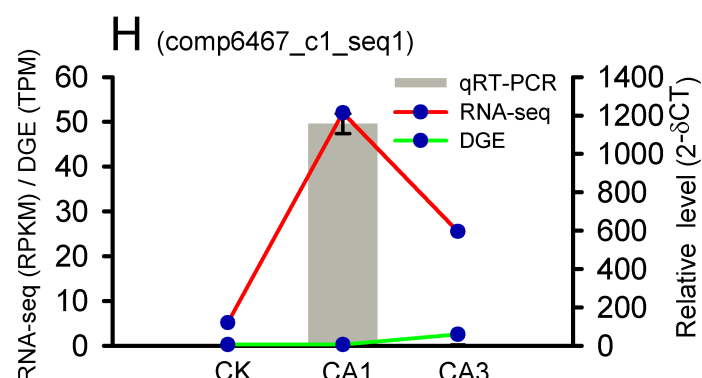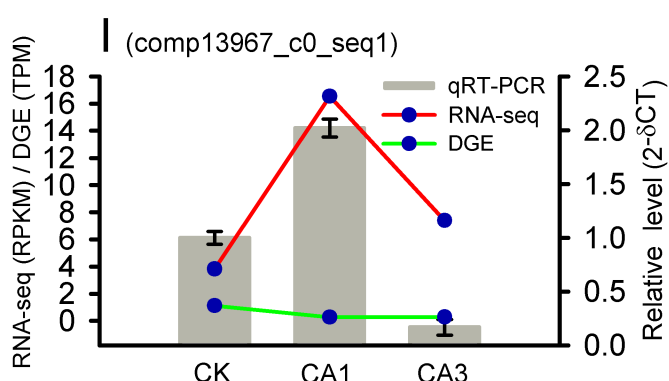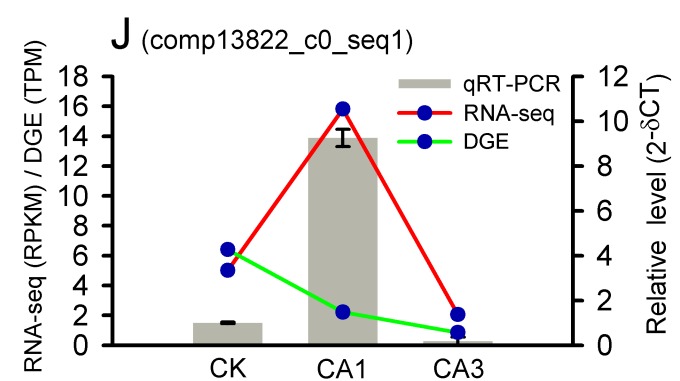

Supplement: Additional file 4 — Quantitative RT-PCR validations for 10 randomly selected transcripts. The quantitative RT-PCR experiment was performed on 10 randomly selected transcripts that show distinct results from RNA-Seq and DGE approaches. Consistent expression patterns between RNA-Seq and qRT-PCR results were observed in eight of these transcripts (A, B, C, D, E, H, I and J), while the other 2 transcripts were only partially consistent (F and G). [file 1471-2164-14-415-S4.pdf]
